# Comparative amino acid decomposition analysis of potent type I p38α inhibitors

**DOI:** 10.1186/2008-2231-21-41

**Published:** 2013-05-29

**Authors:** Ahmad Ebadi, Nima Razzaghi-Asl, Mehdi Khoshneviszadeh, Ramin Miri

**Affiliations:** 1 Medicinal and Natural Products Chemistry Research Center, Shiraz University of Medical Sciences, PO Box 3288-71345, Shiraz, Iran; 2 Department of Medicinal Chemistry, School of Pharmacy, Shiraz University of Medical Sciences, PO Box 1583-71345, Shiraz, Iran

**Keywords:** p38α, Anti-inflammatory, Amino acid decomposition analysis, DFT, Molecular dynamic

## Abstract

**Background and purpose of the study:**

p38α is a member of mitogen-activated protein kinases (MAPK) considered as a prominent target in development of anti-inflammatory agents. Any abnormality in the phosphorylation process leads to the different human diseases such as cancer, diabetes and inflammatory diseases. Several small molecule p38α inhibitors have been developed up to now. In this regard, structural elucidation of p38 inhibitors needs to be done enabling us in rational lead development strategies.

**Methods:**

Various interactions of three potent inhibitors with p38α active site have been evaluated in terms of binding energies and bond lengths via density function theory and MD simulations.

**Results:**

Our comparative study showed that both *ab initio* and MD simulation led to the relatively similar results in pharmacophore discrimination of p38α inhibitors.

**Conclusion:**

The results of the present study may find their usefulness in pharmacophore based modification of p38α inhibitors.

## Introduction

In the process of drug discovery, lead generation is a key bottleneck. The costly experimental testing of so many compounds leads to a real challenge in high throughput screening (HTS) method and makes it critical to perform virtual screening techniques to reduce the size of chemical collection richen in active compounds [1]. Computer based prescreening of chemical databases has found its key role in lead identification and is known as *in silico* drug design. Generally speaking, *in silico* drug design falls into four categories that are related to each other depending on the structural information on targets and their ligand. These methods are structure-based design, ligand-based design, combinatorial chemistry-based design and de novo design [2].

De novo design techniques are used in the case of known receptor structure and unknown ligand structure. One of the most efficient and rational methods to afford this challenge is fragment based drug design [3,4]. In fragment based drug design, binding of small molecule fragments (MW < 300, No. H-donors < 3, No. H-acceptors < 3, ClogP < 3) [5] to specific domain of active site is evaluated. Based on the binding energies, best fragments are selected and bridged together with appropriate linker(s) to generate new scaffolds. The reverse process, *i.e.* fragmentation of ligands to constructing fragments, can be used for modification of known ligands. By fragmentation, the chemical diversity of fragment database decreases and the chance of achievement to new lead compound increases. In this process, assessment of interaction between fragments and receptor is the rate limiting step. Estimating the contribution of individual amino acid-ligand interaction energies in total binding energy, *i.e.* Amino acid Decomposition Analysis (ADA) [6-8], would be a very useful trend in fragment development. ADA is based on receptor structure and could be applied to different types of scaffolds. The power of ADA in predicting the effect of individual residues on ligand-receptor interactions can be used as supporting information in drug design. In this regard, estimation of the optimum binding geometry could assist in choosing the best fragment(s) leading to the improved ligand potency profiles.

The phosphorylation of proteins by protein kinases, the largest family of signaling proteins, regulates cell life. More than 500 protein kinases are encoded by the human genome and it is no surprise that any abnormality in the phosphorylation process would lead to the different human diseases such as cancer, diabetes and inflammatory diseases. Different types of these regulating enzymes are introduced as therapeutic target. The active site conservation between protein kinases makes it a real challenge to design selective agents [9-12]. Therefore evaluation of structural features of these protein kinases and the role of fragments to achieve selectivity may be regarded as an important topic.

p38 belongs to the family of mitogen-activated protein kinases (MAPK). p38 MAPKs are generally divided to various isoforms including α, β, γ and δ types [13,14]. p38α and p38β are vital biological targets in inflammatory pathways [15]. MAP kinase kinase3 and 6 (MKK3, 6) are activated by inflammatory factors such as IL-1, TNFα and cell stress [16-18]. MKK3 and 6 are upstream kinases that phosphorylate the tyrosine and threonine residues in p38α and hence activate it [19]. The activated p38α stimulates the IL-1, TNFα and COX-2, enhances the transcription of inflammatory genes, and also has been found to stabilize the inflammatory response protein mRNAs [20,21].

**Figure 1:**
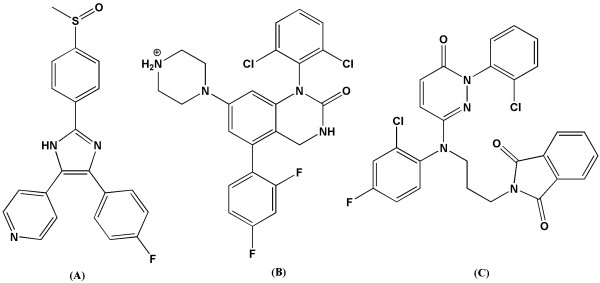
**Structure of diarylimidazole (SB203580) IC**_**50**_**: 4.8 nM (A), dihydroquinazolinone IC**_**50**_**: 2.6 nM (B) and 2-arylpyridazin-3-one IC**_**50**_**: 5 nM (C) p38α type &Iota; inhibitors.**

Considering the important role in inflammatory pathways, p38 can be regarded as an attractive target to design and develop anti-inflammatory agents. Indeed, p38α is a distinguished target in development of anti-inflammatory agents. Different classes of p38α inhibitors have been developed up to now and their pharmacophore were evaluated in detail [22-27]. In the present contribution, we used MD simulations and *ab initio* method to evaluate pharmacophore model of three potent type &Iota; p38α inhibitors comprehensively. The results of both MD and *ab initio* methods were reported and compared with each other. Three different inhibitors, diarylimidazole [28], dihydroquinazolinone [29] and 2-arylpyridazin-3-one [30] scaffolds were selected for our study (Figure 1). These inhibitors are direct ATP-binding site inhibitors with sub-micromolar to nanomolar activity. **SB203580** inhibits p38α and β with almost similar potency. This compound is ~10 times selective towards p38α/β compared to p38γ/δ [31].

**Figure 2:**
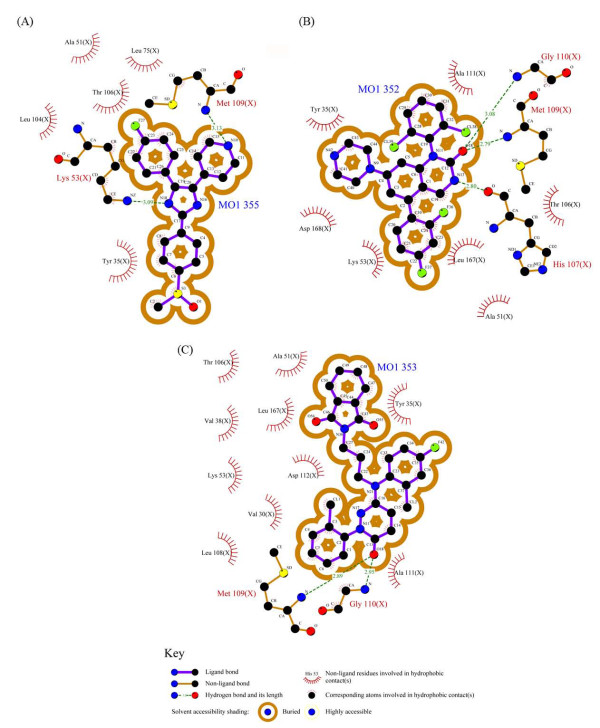
2D schematic representation of interactions between SB203580 (A), dihydroquinazolinone (B) and 2-arylpyridazin-3-one (C) scaffolds and p38α active site generated by LIGPLOT.

In the case of **SB203580**, crystallographic studies (PDB code 1A9u, resolution: 2.50 Ǻ) demonstrated that pyridyl nitrogen formed a hydrogen bond with Met109 (Figure 2). Moreover; 4-fluorophenyl ring occupied the hydrophobic pocket adjacent to the Met109. These two types of interactions have been observed in most of the ATP-binding inhibitors [32]. Nitrogen atom of imidazole ring interacts with Lys53 via hydrogen bond and electrostatic forces. Electrostatic forces are long range interactions between ligand and receptor and have determinant effect on ultimate ligand-receptor complex stability.

For dihydroquinazolinone (PDB code: 1M7Q, resolution: 2.40 Ǻ) and 2-arylpyridazin-3-one (PDB code: 2I0H, resolution: 2.00) scaffolds, the same pattern of binding in the p38α active site have been reported (Figure 2) [29,30]. Both of these inhibitors have a carbonyl moiety that interacts with Met109 and Gly110 backbone NH via hydrogen bonds. 2,4-diflourophenyl and 2-chloro-4-flouropheny moieties in dihydroquinazolinone and 2-arylpyridazin-3-one inhibitors occupied the hydrophobic pocket in the proximity of Met109. Dihydroquinazolinone scaffold has an additional hydrogen bond with His107 and 2-arylpyridazin-3-one has more hydrophobic interactions when compared with dihydroquinazolinone.

In this project we used *ab initio* method and MD simulations to pharmacophore modeling of these three potent inhibitors via ADA strategy. B3LYP functional [33] and BP86 functional [34] together supplemented with triple-ζ basis set (TZV) [35] were used with the aim of:

Evaluation of ligand-p38α stability via molecular dynamic simulations

ADA through MD simulations and *ab initio* method to detect hot spots in p38α active site

Comparative evaluation of MD and *ab initio* results

## Materials and methods

### Preparation of data set

X-ray crystallographic structures of p38α with its cognate ligands were obtained from Brook Haven Protein databank (http://www.rcsb.org). The Swiss PDB Viewer program (http://www.expasy.org/spdbv) [36] was used to rebuilt and add missed atoms of Lys15 and Arg173 in 1M7Q and 2I0H PDB structures. The crystallographic *holo* structures were used as a starting point for MD simulations. The force field parameters of ligands were obtained using PRODRG, an automated topology generation tool web server [37]. The electrostatic potential (ESP) derived charges of ligands were all recalculated according to Breneman and Wiberg [38] by using B3LYP/TZV(2d,2p) method and were adjusted in topology file.

The evaluated amino acid residues were selected on the basis of information from schematic 2D representations of ligand-receptor interactions produced by LIGPLOT program [39].

In *ab initio* studies, all amino acids were considered in their real electrostatic state. Each residue under study was truncated at the C-terminal and N-terminal. N-terminal was acetylated and C-terminal was methyl amidated to mimic the original electron density profile. All conformational and configurational features were the same as the X-ray structure. Molecular images were produced using VMD program [40].

### Molecular dynamics simulations

Molecular dynamics simulations were carried out using GROMACS 4 package with the standard GROMOS96 force field [41]. In each case, the p38-ligand complex was solvated in a cubic box with the dimension of 95 × 95 × 95 (all in Ǻ). Explicit simple point charge model (SPC216) was used to represent water molecules [42]. Na^+^ atoms were added to neutralize the total charge of the systems. Short-range interactions were evaluated using a twin-rang cutoff with van der Waals and electrostatic interactions truncated at 12 and 10 Ǻ, respectively. Particle mesh Ewald (PME) method was used to evaluate long-range electrostatic interactions. The protein-ligand complex and waters along with ions were coupled in a temperature bath at 300K, separately (τ_T_ = 0.1 ps). Berendsen barostat (τ_p_ = 1 ps) was used to maintain pressure in 1.0 atm [43,44]. Linear constraint solver (LINCS) was applied to constrain all bonds [45].

In the first step, energy minimization was performed using steepest descent integrator realized in GROMACS package. After energy minimization, 100 ps NVT and NPT ensembles were used to equilibrate system. During NVT and NPT ensembles a harmonic position restrain (1000 kJ mol^-1^ nm^-1^) was applied to all the heavy atoms of the p38α and ligand. Following equilibration step, production of MD simulation was conducted for 20 ns without any constrains. Finally, analysis programs included in GROMACS package were used to evaluate trajectories.

### *Ab initio* method

All calculations were performed on a structure that obtained by averaging over last 10 ns of MD simulations. Heavy atom-hydrogen bonds were optimized by using heavy atom fixing approximation (constrained optimizations). Constrained optimization was used to prevent irrational movement of the side chains. Otherwise the position of ligand and relevant residue could be changed drastically. Neese and Bykov evaluated optimization errors of this type [46]. According to their evaluation, obtained results are reliable. BP86 functional together in association with triple-ζ basis set (TZV) was used in optimization process. Resolution-of-identity (RI) approximation [47] together with fitting auxiliary basis set TZV/J [48] was applied for all atoms.

Energy calculations were done using B3LYP (Becke-Lee-Yang-Parr) functional in association with triple-ζ basis set (TZV) on optimized structures. For these calculations, chain-of-spheres (RIJCOSX) approximation [49] was invoked. Two set of first polarization functions were applied on hydrogen and non-hydrogen atoms [TZV(2d,2p)]. To consider long-range dielectric effect of protein in our calculation, COSMO model [50] with a dielectric constant of 4.8 was applied [51]. All calculations were done using the ORCA quantum chemistry package [52].

Ligand-residue binding energies (ΔE_b_) were calculated using the previously introduced equation [53]. Counterpoise correction [54] was used to take into account basis set superposition error (BSSE). In the case of **SB203580**, Potential Energy Surface (PES) scan were performed in the direction of hydrogen bond with Met109 in forty steps considering 0.05Å step sizes. The PES calculations were performed by the same method and basis set as mentioned above.

## Results and discussion

### MD simulations

Crystallographic structure of p38α with its cognate ligands enabled us to perform MD simulations and evaluate the role of individual amino acids in total binding energy. This structure was used as starting conformation for our simulations. In the first step, we performed a 20 ns MD simulation to reach a stable trajectory.

**Figure 3:**
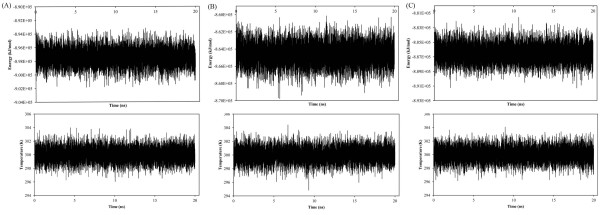
Total energy and temperature variations during 20 ns MD simulation; SB203580 (A), dihydroquinazolinone (B) and 2-arylpyridazin-3-one (C).

**Figure 4:**
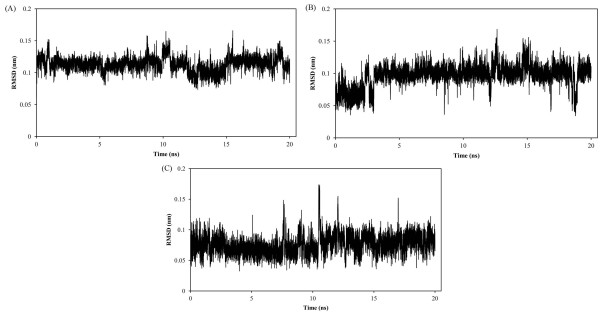
RMSD of ligand atoms; SB203580 (A), dihydroquinazolinone (B) and 2-arylpyridazin-3-one (C).

The stabilities of trajectories were confirmed by assessment of total energy, temperature and RMSD (Figures 3 and 4).

The Average temperature during 20 ns MD simulation at 300.0K was found to be 300K (±1.32, 1.34 and 1.33 respectively) for these systems (Figure 3). These results

 represented that the obtained equilibriums and energy conservations for the studied systems were desirable.

The RMSDs of ligands in the active site of p38α with respect to their initial structures were used to evaluate the stabilities of these three complexes.

In the case of **SB203580** (Figure 4A), RMSD rose up to 1.13 Ǻ at the beginning of MD simulations and fluctuated around this value (± 0.15) for the rest of simulation. This distribution pattern demonstrated us that ligand achieved to the equilibrium state just after 1 ns distinguished by the RMSD profile. For dihydroquinazolinone scaffold (1M7Q), RMSD increased to 0.71 Ǻ and leveled off to nearly 3 ns (Figure 4B). At this point of simulation, RMSD rose up again to 1 Ǻ and leveled off (± 0.13). In the case of 2-arylpyridazin-3-one scaffold, RMSD increased to 0.76 Ǻ and fluctuated around (± 0.16) over the course of MD simulation time (Figure 4C). These data showed that evaluated ligands reached to an equilibrium state after preliminary fluctuations.

**Figure 5:**
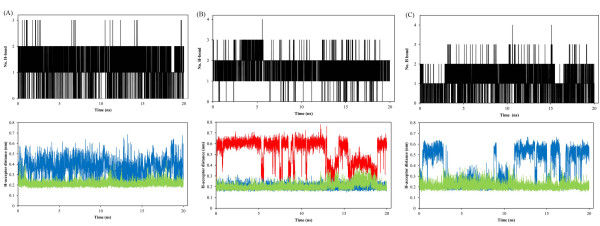
Number and distance of H-bonds between ligands and p38α active site residues; SB203580 (A), dihydroquinazolinone (B) and 2-arylpyridazin-3-one (C).

During MD simulations the average of 1.2 H-bond(s) could be detected between **SB203580** and p38α active site residues (Figure 5A). The variation of donor-acceptor

 distance during MD simulation could be used to evaluate the forming and breaking of H-bonds. Over the whole process, the donor-acceptor distances less than 3.5 Ǻ demonstrated hydrogen bond formation. As can be seen in Figure 5A, pyridine nitrogen-Met109 NH distance remained less than 3 Ǻ for the whole simulation time (green). This fact could convince us in considering a permanent H-bond between these two moieties. But hydrogen bond between imidazole nitrogen and quaternary amine hydrogen of Lys53 was significantly less detectable. This hydrogen bond was formed and broken frequently during MD simulations (Figure 5A blue).

In the case of dihydroquinazolinone scaffold, an average of 1.5 H-bond(s) with p38α active site residues (Figure 5B) could be detected. Results showed that H-bond between Met109 NH and ligand O18 atoms (green) existed during whole MD simulation period. The distance between His107 O and ligand HN13 atoms remained less than 3.5 Ǻ during MD simulation (blue). According to obtained results, these two hydrogen bonds are permanent during 20 ns MD simulations. The Gly110 NH-ligand O18 distance (red) fluctuated between 5 ns and 20 ns. However the averaged distance remained higher than 0.35 Ǻ for 98.8% of the simulation period (Temporary H-bond, Table 1).

2-arylpyridazin-3-one scaffold formed an average of 1.2 H-bond(s) with Met109 and Gly110 during MD simulation (Figure 5C). In this case, the distance between Met109 NH and ligand O18 atoms was almost under 0.35 Ǻ in the whole period (green). But the distance between Gly110 NH and ligand O18 atoms was higher than 0.35 Ǻ in 49.5% of simulation period (blue). These outcomes showed that Met109-ligand and Gly110-ligand H-bonds were of permanent and temporary types, respectively.

On the basis of results (Table 1), it might be concluded that hydrogen bond between ligand and Met109 is the key structural point in binding to the receptor. This interaction is the common structural feature of all type &Iota; p38α inhibitors. More detailed analysis of H-bonds between p38α active site residues and evaluated ligands is summarized in Table 1.

**Figure 6:**
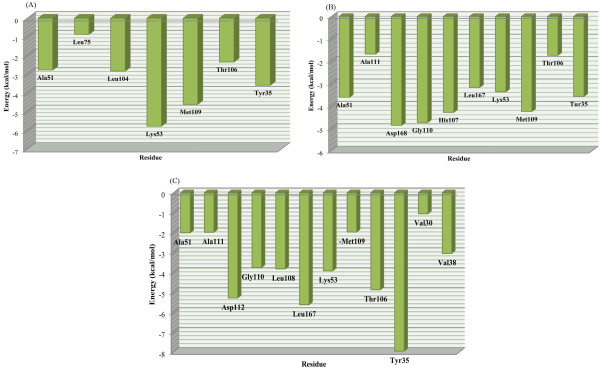
Contribution of each residue of p38α in binding to SB203580 (A), dihydroquinazolinone (B) and 2-arylpyridazin-3-one (C) scaffolds (calculated by MD simulations).

After obtaining an equilibrium system, ADA was carried out as follow: participation of each amino acid in total binding energy was computed by evaluation of Lennard-Jones (LJ) and coulombic interaction energies between each amino acid and ligands via performing an additional 1 ns MD simulation in each case. The results of ADA are shown in Figure 6 and Table 2. Negative energy shifts showed that the residue made favorable contribution to ligand-receptor interactions.

LIGPLOT program [39] was used to detect residues that interact with ligand in each case. Based on the obtained data, same binding pattern to p38α active site could be detected in all the scaffolds. Interaction energies with hinge region residues (Met109, Gly110, and Ala111) are significant and in each case at least, there is one interaction with these amino acids. Residues constructing hydrophobic pocket in the proximity of Met109 were almost involved in interactions with ligand.

In **SB203580**, Lys53 was found to be the most significant residue in ligand-receptor interactions (ΔE: -5.77 kcal/mol). Nitrogen atom of an imidazole ring participated in H-bond with quaternary amine hydrogen of Lys53. In fact electrostatic forces between these groups made it a favorable interaction. Lys53 had maximum coulombic and LJ interaction energies in these series (-1.64 and -4.13 kcal/mol, respectively). Electrostatic interactions are important forces in primary approach of ligand and receptor to each other. These types of interactions are of long-range type

**Table 1:** Hydrogen bond analysis between evaluated ligands and p38α residues

**System**	**Donor**	**Acceptor**^**1**^	**H-bond distance**^**2 **^**( Ǻ)**	**H-bond angle**^**3 **^**(degree)**	**Occupancy (%)**
1A9U	Met109 NH	Ligand N10	2.14 ( ± 0.24)	14.2 ( ± 8.78)	99.8
	Lys53 HZ	Ligand N18	3.66 ( ± 0.85)	58.5 ( ± 23.7)	39.7
1M7Q	Met109 NH	Ligand O18	2.06 ( ± 0.32)	14.4 ( ± 11.4)	99.8
	Gly110 NH	Ligand O18	5.30 ( ± 1.15)	55.1 ( ± 29.0)	11.2
	Ligand HN13	His107 O	1.98 ( ± 0.20)	14.4 ( ± 8.10)	100
2I0H	Met109 NH	Ligand O18	2.09 ( ± 0.28)	23.9 ( ± 14.8)	99.8
	Gly110 NH	Ligand O18	3.81 ( ± 1.58)	38.2 ( ± 28.5)	50.5

^1^Atom numbers are according to Figure 2.

^2^The average H-bond distance with standard deviation in parentheses.

^3^The average H-bond angle with standard deviation in parentheses.

 and determinative in the final ligand-receptor complex stability. According to the obtained results, imidazole ring (interacting with Lys53) is a very important moiety in diarylimidazole based p38α inhibitors.

Met109 backbone hydrogen formed a hydrogen bond with pyridine nitrogen (ΔE: -4.61 kcal/mol). Hydrogen bond with hinge region residue (Met109) is the key feature of ATP binding site (type &Iota;) inhibitors and could be observed in all type &Iota; inhibitors. Accumulated negative charge on pyridine ring of **SB203580** formed a favorable interaction (hydrogen bond and electrostatic forces) with Met109.

Ala51, Leu75, Leu104 and Thr106 contributed to important hydrophobic contacts in the hydrophobic pocket (ΔE: -2.76, -0.87, -2.83 and -2.34 kcal/mol, respectively). These hydrophobic interactions had minimum coulombic interaction energies (Table 2). Due to the reported pharmacophore models of diverse classes of p38 MAPK [22], interactions with Met109 and this hydrophobic pocket are the chemical features designated for type &Iota; p38α inhibitors. Tyr35 participated in π-π stacking interaction with *para*-methylsulfinyl phenyl ring of **SB203580** (ΔE: -3.59 kcal/mol).

In the case of dihydroquinazolinone scaffold (1M7Q), His107 (-4.28 kcal/mol), Met109 (-4.24 kcal/mol), Gly110 (-4.72 kcal/mol) and Asp168 (-4.86 kcal/mol) residues had maximum binding energies. His107, Met109 and

**Table 2:** LJ and coulombic interaction energies (kcal/mol) between p38α active site residues and evaluated ligands

**1A9U**			**1M7Q**			**2I0H**		
**Residue**	**Coul**	**LJ**	**Residue**	**Coul**	**LJ**	**Residue**	**Coul**	**LJ**
Ala51	-0.23	-2.53	Ala51	-0.39	-3.21	Ala51	-0.05	-1.95
Leu75	0.00	-0.87	Ala111	0.11	-1.79	Ala111	0.08	-2.06
Leu104	-0.48	-2.35	Asp168	-3.56	-1.30	Asp112	-1.61	-3.66
Lys53	-1.64	-4.13	Gly110	-2.09	-2.63	Gly110	-1.31	-2.44
Met109	-2.87	-1.74	His107	-3.56	-0.72	Leu108	-0.32	-3.49
Thr106	0.10	-2.44	Leu167	0.00	-3.15	Leu167	0.01	-5.62
Tyr35	0.11	-3.70	Lys53	0.69	-4.04	Lys53	0.21	-4.11
			Met109	-2.23	-2.01	Met109	-0.58	-1.39
			Thr106	0.12	-1.85	Thr106	-1.48	-3.37
			Tyr35	-1.15	-2.41	Tyr35	-0.07	-7.86
						Val30	0.01	-1.06
						Val38	0.00	-3.04

 Gly110 interact via hydrogen binding and Asp168 interact via electrostatic interactions (maximum coulombic interaction -3.56 kcal/mol). Lys53 had minimum coulombic interaction energy (0.69 kcal/mol) due to nearness of Lys53 quaternary amine to positive N42 atom in this ligand.

2-arylpyridazin-3-one scaffold (2I0H) had maximum biding energy with Tyr35. Our model indicated that Isoindoline-1,3-dione ring interacted with Tyr35 via π-π stacking. This interaction was associated with maximum LJ interaction energy (-7.86 kcal/mol). Met109 (-1.97 kcal/mol) and Gly110 (-3.75 kcal/mol) backbone NHs interacted with ligand O18 atom via H-bond. This ligand had more hydrophobic interactions in comparison with previous ones. LJ and coulombic interaction energies in each case were summarized in Table 2.

By using ADA we could model the binding mode of three different p38α inhibitors. Obtained results were in good agreement with evaluated pharmacophore models in the literature [22].

### *Ab initio* evaluation

In this part we used *ab initio* method to evaluate contribution of individual amino acid-ligand interaction energies in total binding energy and compare obtaining results with MD simulations.

The constrained optimization process was done using BP86/TZV method on a structure which was obtained by averaging over last 10 ns MD simulations. All *ab initio* studies were done on achieved optimized structure.

**Figure 7:**
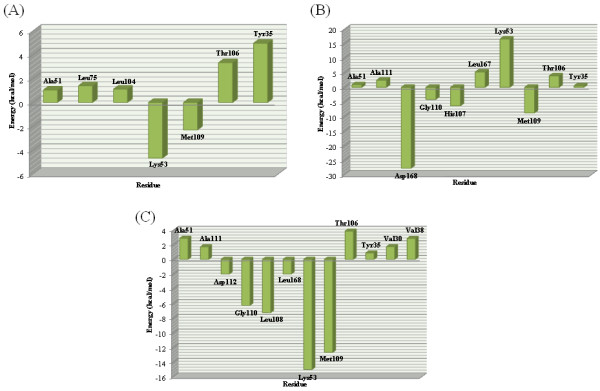
**Contribution of each residue of p38α in binding to SB203580 (A), dihydroquinazolinone (B) and 2-arylpyridazin-3-one (C) scaffolds (calculated by *ab initio*).**

Various interaction energies between studied p38α inhibitors and selected residues in the active site were obtained independently. The relevant data are shown in Figure 7.

Interactions between imidazole nitrogen and quaternary amine of Lys53 in **SB203580** had the most considerable interaction energy (-4.66 kcal/mol) (Figure 7A). This strong interaction occurred as a result of electrostatic forces between positive nitrogen (cationic Lys53, atomic partial charge: +0.51) and partially negative imidazole N1 atom (atomic partial charge: -0.62). This ionic dipole interaction had determinant participation in total ligand-receptor binding energy.

Another important interaction could be recorded between Met109 and pyridine nitrogen (-2.30 kcal/mol). Interestingly, residues participated in hydrophobic interactions exhibited repulsive interaction with evaluated inhibitor. In the case of Tyr35, the repulsive interaction may be interpreted on the basis of inappropriate orientation of ligand *para*-methylsulfinyl phenyl ring versus Tyr35 phenyl ring. It should be noted, p38α inhibitors lacking this moiety might not have any significant effect on ligand potency [28].

Asp168 carboxylic moiety interacts via electrostatic forces with quaternary amine in dihydroquinazolinone ligand. This major interaction had prominent binding energy in this series of residues. Hydrogen bond between Met109 backbone NH and ligand O18 atom had binding energy equal to -8.78 kcal/mol. Negative binding energies could be detected between His107 backbone NH and HN18 (-6.34 kcal/mol), Gly110 backbone NH and ligand O18 atom (-4.16 kcal/mol) but like the other ones all hydrophobic interactions had positive contribution in binding energy. Proximity of Lys53 and ligand quaternary amines made this interaction inefficient.

In the case of 2-arylpyridazin-3-one scaffold, Cation-π interaction could be detected between Lys53 and 4-flouro-2-methilphenyl moiety. This interaction had maximum binding energy. Hydrogen binding could be

 detected between Met109 and Gly110 backbones NH and ligand O18 atom (-12.66 and -6.27 kcal/mol, respectively).

Owing to the important role of hydrogen bond with Met109 in type &Iota; inhibitors, we decided to optimize the geometric position of the involving functional group in the **SB203580** ligand. For this purpose, hydrogen bond distance between Met109 backbone hydrogen and pyridyl nitrogen in **SB203580** was scanned in the original direction. The maximum interaction energy was found in the 2.25 Å bond length (data was not shown). The optimum distance between pyridine nitrogen and Met109 backbone hydrogen after 20 ns MD simulation was calculated to be 2.14 Å. These findings interestingly showed that *ab initio* method and MD simulations converged to the same results. Moreover, it was demonstrated that crystallographic structures may not be appropriate starting points for *ab initio* calculations in all cases.

### Comparison of the two methods

MD simulations and *ab initio* methods were used to calculate the involvement of each amino acid in total binding energy. The results of applied methods were compared to reveal the accuracy and efficiency levels. Our calculations revealed that MD simulations and *ab initio* based studies led to the similar trends in estimation of amino acid-ligand binding energies. In both methods residues responsible for major interactions in the p38α active site could be recognized with adaptable level of reproducibility (Figures 6 and 7).

**Figure 8:**
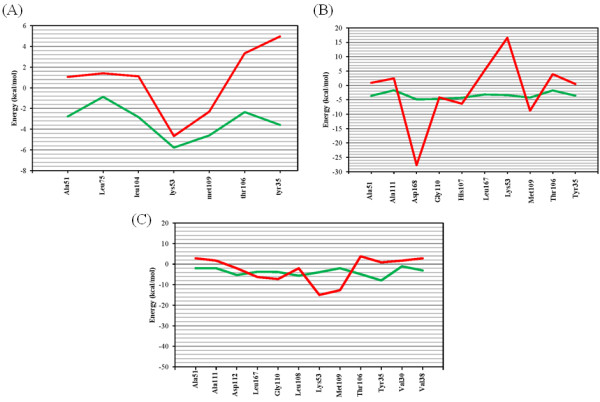
**Comparative results of MD simulations (green) and *ab initio *studies (red) in SB203580 (A), dihydroquinazolinone (B) and 2-arylpyridazin-3-one (C) scaffolds.**

For p38α active site, *ab initio* method resulted in more repulsive hydrophobic and more attractive electrostatic interactions when compared to MD simulations (Figure 8). This effect seemed to be probably related to the solvent effect and also interactions among adjacent residues. Moreover B3LYP method tended to produce more polarized wave function in electrostatic interactions [55] leading to false positive values.

For instance in p38α, Lys53 interacted with Asp168 and this electrostatic interaction decreased the attractive interaction between Lys53 and **SB203580** in MD simulations. But in *ab initio* study, just the interaction between Lys53 (truncated at N-terminal and C-terminal) and ligand was considered. Similar binding patterns for nearly all residues could be detected while in the case of charge-assisted interactions (Lys53, Asp168), significant deviations were seen (Figure 8). However, relatively similar

 binding energies were estimated for Lys53 in **SB203580**. Two rationales might be envisaged for this different trend:

Solvent effects were attended in MD simulations while *ab initio* studies were performed in vacuum

In **SB203580**, Lys53 interacted with partially negative imidazole sp^2^ nitrogen atom

Probable repulsive forces between dihydroquinazolinone piperazinium and Lys53 quaternary amine

2-arylpyridazin-3-one scaffold contributed in cation-π interaction with Lys53

## Conclusion

We applied totally 60 ns MD simulations and *ab initio* method to evaluate and compare the accuracy of these methods in predicting pharmacophore models of three different p38α MAPK inhibitors. Both methodologies were able to unravel key interactions with different p38α inhibitors. One advantageous feature of DFT-based calculations is their relatively adaptable outputs regarding significantly shorter processing times due to the incorporated approximations. Results indicated that LJ interactions contributed significantly to binding of **SB203580**, dihydroquinazolinone and 2-arylpyridazin-3-one scaffolds despite the important role of electrostatic interactions in initial approach of ligands to the receptor. We used enzyme structure that was obtained by averaging over last 10 ns of MD simulations for our *ab initio* studies. This technique led to the results indicating that crystallographic primary structures may be used with care in such calculations. Further investigations would be required to rule out widespread structure activity relationships of p38α inhibitory activity. Finally the results of the present study may find their usefulness in pharmacophore based modification of p38α inhibitors.

## Competing interests

The authors declare that they have no competing interests.

## Authors’ contributions

AE: Molecular Dynamics, NR-A: ab Initio, MK: ab Initio, RM: Molecular Dynamic, ab Initio, All authors read and approved the final manuscript.

## References

[B1] Leach AR Hann MM The in silico world of virtual libraries Drug Discov Today 2000 5 326 336 10.1016/S1359-6446(00)01516-6 10893546

[B2] Foye WO Lemke TL Williams DA Foye's principles of medicinal chemistry 2007 Lippincott Williams & Wilkins

[B3] Warr WA Fragment-based drug discovery J Comput Aided Mol Des 2009 23 453 458 10.1007/s10822-009-9292-1 19554264

[B4] Erlanson DA McDowell RS O'Brien T Fragment-based drug discovery J Med Chem 2004 47 3463 3482 10.1021/jm040031v 15214773

[B5] Congreve M Carr R Murray C Jhoti H A'rule of three'for fragment-based lead discovery? Drug Discov Today 2003 8 876 14554012 10.1016/s1359-6446(03)02831-9

[B6] Hensen C Hermann JC Nam K Ma S Gao J Höltje HD A combined QM/MM approach to protein-ligand interactions: polarization effects of the HIV-1 protease on selected high affinity inhibitors J Med Chem 2004 47 6673 6680 10.1021/jm0497343 15615516

[B7] Thangapandian S John S Lee KW Molecular Dynamics Simulation Study Explaining Inhibitor Selectivity in Different Class of Histone Deacetylases J Biomol Struct Dyn 2012 29 677 698 10.1080/07391102.2012.10507409 22208272

[B8] de Brito MA Rodrigues CR Cirino JJV Araújo JQ Honório T Cabral LM de Alencastro RB Castro HC Albuquerque MG Residue-Ligand Interaction Energy (ReLIE) on a Receptor-Dependent 3D-QSAR Analysis of S-and NH-DABOs as Non-Nucleoside Reverse Transcriptase Inhibitors Molecules 2012 17 7666 7694 10.3390/molecules17077666 22732882 PMC6269006

[B9] Noble MEM Endicott JA Johnson LN Protein kinase inhibitors: insights into drug design from structure Science Signalling 1800 2004 303 10.1126/science.1095920 15031492

[B10] Weinmann H Metternich R Editorial: drug discovery process for kinease inhibitors ChemBioChem 2005 6 455 459 10.1002/cbic.200500034 15742380

[B11] Davies SP Reddy H Caivano M Cohen P Specificity and mechanism of action of some commonly used protein kinase inhibitors Biochem J 2000 351 95 10.1042/0264-6021:3510095 10998351 PMC1221339

[B12] Bain J McLauchlan H Elliott M Cohen P The specificities of protein kinase inhibitors: an update Biochem J 2003 371 199 10.1042/BJ20021535 12534346 PMC1223271

[B13] Saklatvala J The p38 MAP kinase pathway as a therapeutic target in inflammatory disease Curr Opinin Pharmacol 2004 4 372 377 10.1016/j.coph.2004.03.009 15251131

[B14] Chen Z Gibson TB Robinson F Silvestro L Pearson G Xu B Wright A Vanderbilt C Cobb MH MAP kinases Chem Rev 2001 101 2449 2476 10.1021/cr000241p 11749383

[B15] Hale KKTD Rihanek M Manthey CL Differential expression and activation of p38 mitogen-activated protein kinase alpha, beta, gamma, and delta in inflammatory cell lineages J Immunol 1999 162 4246 4252 10201954

[B16] Han J Lee JD Jiang Y Li Z Feng L Ulevitch RJ Characterization of the structure and function of a novel MAP kinase kinase (MKK6) J Biol Chem 1996 271 2886 2891 10.1074/jbc.271.6.2886 8621675

[B17] Derijard B Raingeaud J Barrett T Wu IH Han J Ulevitch RJ Davis RJ Independent human MAP-kinase signal transduction pathways defined by MEK and MKK isoforms Science 1995 267 682 685 10.1126/science.7839144 7839144

[B18] Schieven GL The p38 kinase plays a central role in inflammation Curr Top Med Chem 2009 9 1038 1048 10.2174/156802609789630974 19747121

[B19] Enslen H Raingeaud J Davis RJ Selective activation of p38 mitogen-activated protein (MAP) kinase isoforms by the MAP kinase kinases MKK3 and MKK6 J Biol Chem 1998 273 1741 1748 10.1074/jbc.273.3.1741 9430721

[B20] Kracht M Saklatvala J Transcriptional and post-transcriptional control of gene expression in inflammation Cytokine 2002 20 91 106 10.1006/cyto.2002.0895 12453467

[B21] Clark AR Dean JLE Saklatvala J Post-transcriptional regulation of gene expression by mitogen-activated protein kinase p38 FEBS Lett 2003 546 37 44 10.1016/S0014-5793(03)00439-3 12829234

[B22] Sarma R Sinha S Ravikumar M Kishore Kumar M Mahmood S Pharmacophore modeling of diverse classes of p38 MAP kinase inhibitors Eur J Med Chem 2008 43 2870 2876 10.1016/j.ejmech.2008.02.014 18406015

[B23] Wrobleski ST Doweyko AM Structural comparison of p38 inhibitor-protein complexes: a review of recent p38 inhibitors having unique binding interactions Curr Top Med Chem 2005 5 1005 1016 10.2174/1568026054985894 16178743

[B24] Karcher SC Laufer SA Successful structure-based design of recent p38 MAP kinase inhibitors Curr Top Med Chem 2009 9 655 676 10.2174/156802609789007363 19689372

[B25] Pettus LH Wurz RP Small molecule p38 MAP kinase inhibitors for the treatment of inflammatory diseases: novel structures and developments during 2006–2008 Curr Top Med Chem 2008 8 1452 1467 10.2174/156802608786264245 18991731

[B26] Hynes J Leftheris K Small molecule p38 inhibitors: novel structural features and advances from 2002–2005 Curr Top Med Chem 2005 5 967 985 10.2174/1568026054985920 16178741

[B27] Bolos J Structure-activity relationships of p38 mitogen-activated protein kinase inhibitors Mini Rev Med Chem 2005 5 857 868 10.2174/1389557054867048 16178727

[B28] Wang Z Canagarajah BJ Boehm JC Kassis S Cobb MH Young PR Abdel-Meguid S Adams JL Goldsmith EJ Structural basis of inhibitor selectivity in MAP kinases Structure 1998 6 1117 1128 10.1016/S0969-2126(98)00113-0 9753691

[B29] Stelmach JE Liu L Patel SB Pivnichny JV Scapin G Singh S Hop CECA Wang Z Strauss JR Cameron PM Design and synthesis of potent, orally bioavailable dihydroquinazolinone inhibitors of p38 MAP kinase Bioorg Med Chem Lett 2003 13 277 280 10.1016/S0960-894X(02)00752-7 12482439

[B30] Natarajan SR Heller ST Nam K Singh SB Scapin G Patel S Thompson JE Fitzgerald CE O’Keefe SJ p38 MAP kinase inhibitors. Part 6: 2-Arylpyridazin-3-ones as templates for inhibitor design Bioorg Med Chem Lett 2006 16 5809 5813 10.1016/j.bmcl.2006.08.074 16945533

[B31] Bain J Plater L Elliott M Shpiro N Hastie CJ Mclauchlan H Klevernic I Arthur JSC Alessi DR Cohen P The selectivity of protein kinase inhibitors: a further update Biochem J 2007 408 297 10.1042/BJ20070797 17850214 PMC2267365

[B32] Wagner G Laufer S Small molecular anti cytokine agents Med Res Rev 2006 26 1 62 10.1002/med.20042 16283677

[B33] Becke AD A new mixing of Hartree–Fock and local density‒functional theories J Chem Phys 1993 98 1372 10.1063/1.464304

[B34] Becke AD Density functional calculations of molecular bond energies J Chem Phys 1986 84 4524 10.1063/1.450025

[B35] The TurboMole basis sets can be obtained from [ftp.chemie.uni-‒karlsruhe.de/pub in the directories basen jac]

[B36] Guex N Peitsch MC SWISS‒MODEL and the Swiss‒Pdb Viewer: an environment for comparative protein modeling Electrophoresis 1997 18 2714 2723 10.1002/elps.1150181505 9504803

[B37] Aalten DMF Bywater R Findlay J Hendlich M Hooft R Vriend G PRODRG, a program for generating molecular topologies and unique molecular descriptors from coordinates of small molecules J Comput Aided Mol Des 1996 10 255 262 10.1007/BF00355047 8808741

[B38] Breneman CM Wiberg KB Determining atom-centered monopoles from molecular electrostatic potentials. The need for high sampling density in formamide conformational analysis J Comput Chem 1990 11 361 373 10.1002/jcc.540110311

[B39] Wallace AC Laskowski RA Thornton JM Ligplot—a program to generate schematic diagrams of protein ligand interactions Protein Eng 1995 8 127 134 10.1093/protein/8.2.127 7630882

[B40] Humphrey W Dalke A Schulten K VMD: visual molecular dynamics J Mol Graphics 1996 14 33 10.1016/0263-7855(96)00018-5 8744570

[B41] Van Der Spoel D Lindahl E Hess B Groenhof G Mark AE Berendsen HJC GROMACS: fast, flexible, and free J Comput Chem 2005 26 1701 1718 10.1002/jcc.20291 16211538

[B42] Rivail L Chipot C Maigret B Bestel I Sicsic S Tarek M Large-scale molecular dynamics of a G protein-coupled receptor, the human 5-HT < sub > 4</sub > serotonin receptor, in a lipid bilayer J Mol Struct (THEOCHEM) 2007 817 19 26 10.1016/j.theochem.2007.04.012

[B43] Bussi G Donadio D Parrinello M Canonical sampling through velocity-rescaling arXiv preprint arXiv:08034060 2008 10.1063/1.2408420 17212484

[B44] Berendsen HJC Postma JPM van Gunsteren WF DiNola A Haak J Molecular dynamics with coupling to an external bath J Chem Phys 1984 81 3684 3690 10.1063/1.448118

[B45] Hess B Bekker H Berendsen HJC Fraaije JGEM LINCS: a linear constraint solver for molecular simulations J Comput Chem 1997 18 1463 1472 10.1002/(SICI)1096-987X(199709)18:12<1463::AID-JCC4>3.0.CO;2-H

[B46] Bykov D Neese F Substrate binding and activation in the active site of cytochrome c nitrite reductase: a density functional study J Biol Inorg Chem 2011 16 417 430 10.1007/s00775-010-0739-6 21125303

[B47] Neese F An improvement of the resolution of the identity approximation for the formation of the Coulomb matrix J Comput Chem 2003 24 1740 1747 10.1002/jcc.10318 12964192

[B48] Eichkorn K Treutler O Öhm H Häser M Ahlrichs R Auxiliary basis sets to approximate Coulomb potentials Chem Phys Lett 1995 240 283 290 10.1016/0009-2614(95)00621-A

[B49] Neese F Wennmohs F Hansen A Becker U Efficient, approximate and parallel Hartree–Fock and hybrid DFT calculations. A ‘chain-of-spheres’ algorithm for the Hartree–Fock exchange Chem Phys 2009 356 98 109 10.1016/j.chemphys.2008.10.036

[B50] Klamt A Schüürmann G COSMO: a new approach to dielectric screening in solvents with explicit expressions for the screening energy and its gradient J Chem Soc Perkin Trans 2 1993 5 799 805

[B51] Bykov D Neese F Reductive activation of the heme iron–nitrosyl intermediate in the reaction mechanism of cytochrome c nitrite reductase: a theoretical study J Biol Inorg Chem 2012 17 1 20 10.1007/s00775-011-0818-3 22454108

[B52] Neese F ORCA – an ab initio, Density Functional and Semiempirical program package, Version 2.8.0 2011 University of Bonn

[B53] Razzaghi-Asl N Ebadi A Edraki N Shahabipour S Miri R *Ab initio* modeling of a potent isophthalamide-based BACE-1 inhibitor: amino acid decomposition analysis Med Chem Res 2013 22 3259 3269 10.1007/s00044-012-0277-6

[B54] Boys S Bernardi F The calculation of small molecular interactions by the differences of separate total energies. Some procedures with reduced errors Mol Phys 1970 19 553 566 10.1080/00268977000101561

[B55] Cramer CJ Essentials of computational chemistry: theories and models 2005 Wiley

